# Non-typhoidal *salmonella* contamination along the pork value chain in a rural East African setting: a cross-sectional study

**DOI:** 10.1093/trstmh/trad046

**Published:** 2023-07-25

**Authors:** Cianjo M Gichuyia, Lian F Thomas, Christine Makena, Linnet Ochieng, Peter B Gathura, Joshua O Onono, Eric M Fèvre

**Affiliations:** Department of Public Health, Pharmacology and Toxicology, University of Nairobi, P. O. Box 29053-00625, Kangemi, Kenya; International Livestock Research Institute (ILRI), P.O. Box 30709-00100, Nairobi, Kenya; International Livestock Research Institute (ILRI), P.O. Box 30709-00100, Nairobi, Kenya; Institute of Infection, Veterinary and Ecological Sciences, University of Liverpool, Liverpool L69 3BX, UK; Department of Public Health, Pharmacology and Toxicology, University of Nairobi, P. O. Box 29053-00625, Kangemi, Kenya; International Livestock Research Institute (ILRI), P.O. Box 30709-00100, Nairobi, Kenya; International Livestock Research Institute (ILRI), P.O. Box 30709-00100, Nairobi, Kenya; Department of Public Health, Pharmacology and Toxicology, University of Nairobi, P. O. Box 29053-00625, Kangemi, Kenya; Department of Public Health, Pharmacology and Toxicology, University of Nairobi, P. O. Box 29053-00625, Kangemi, Kenya; International Livestock Research Institute (ILRI), P.O. Box 30709-00100, Nairobi, Kenya; Institute of Infection, Veterinary and Ecological Sciences, University of Liverpool, Liverpool L69 3BX, UK

**Keywords:** food safety, foodborne disease, non-typhoidal *Salmonella*, pork meat, salmonellosis

## Abstract

**Background:**

Non-typhoidal *Salmonella* (NTS) is a serious foodborne pathogen that has previously been isolated from pigs presented for slaughter in a rural pork value chain in western Kenya.

**Methods:**

To understand varying NTS contamination along the value chain we assessed prevalence at slaughter, transport and retail. Suspect isolates from culture were confirmed using matrix-assisted laser desorption-ionization time of flight mass spectrometry.

**Results:**

Prevalence on pig carcasses, meat transportation containers, retailed raw and cooked pork and accompanying side salads was 18.1%, 23.9%, 28.0%, 1.9% and 8.6%, respectively.

**Conclusion:**

NTS contamination is propagated along the pork value chain in rural western Kenya, demonstrating the need for improved hygiene measures to prevent human exposure.

## Introduction

The Institute for Health Metrics and Evaluation estimated that, in 2010, enteric non-typhoidal salmonellosis accounted for 4 847 000 disability-adjusted life years lost and 81 300 deaths worldwide, with disproportionately higher share/burden observed in sub-Saharan Africa.^1^ Pigs are considered among the most significant sources of infection, especially as asymptomatic carriers of non-typhoidal *Salmonella* (NTS), which poses challenges in preventing their entry into the food chain.^2^ A recent study isolated NTS from 12.7% of pigs being presented for slaughter in rural western Kenya, including serotypes that cause human gastroenteritis.^3^ Previous studies in the same region have also identified NTS in diarrheal patients, with Akullian et al. reporting an isolation rate of 12.4% from stool samples collected from 2007 to 2014, and Shapiro et al. reporting a rate of 4% from stool samples collected during 1997 and 1998.^4,5^ Crump et al.^6^ proposed that meat pathways are potential sources of NTS infections in East Africa, and the burden of NTS attributed to pork consumption has been found to be highest in the African sub-regions.^7^

Considering the awareness of the proportion of *Salmonella-*infected pigs entering this value chain, the present study aimed to investigate how the prevalence of contamination changes throughout the various stages of the food system, starting from the dispatch of dressed carcasses from slaughter to the point of retail, where there is contact with pork consumers. The results are an essential prerequisite for quantifying the public health risk associated with this value chain, enabling the design of targeted and efficient mitigation strategies, particularly in anticipation of a significant increase in pork consumption within this region in the coming decades.^8^

## Materials and Methods

The methodology used in this study is outlined briefly here, with additional details available in the supplemental material. A cross-sectional survey was conducted, and simple random sampling was employed to collect samples at each stage of the pork supply chain from slaughter to retail in Busia County, western Kenya. This region was chosen for the study as existing data are already available for contamination at slaughter^3^ and because the informal pork value chain is growing rapidly in this high-density human population.^8^

Sterile, moist gauze swabs were used to sample 144 post-slaughter carcasses (specifically from the ham, back, belly and jowl), as well as 113 meat transportation containers (sampling from three sides and the bottom of each container), each covering a total surface area of 400 cm^2^. During retail, sampling involved requesting pork retailers to package 250-g portions of raw and/or cooked pork as well as vegetable side salads using their regular equipment, as they routinely carry out for their customers. A total of 247 retail raw pork samples from 125 raw pork retail outlets, as well as 104 cooked pork samples and 81 raw vegetable salad samples from 104 cooked pork retail outlets, were collected.

*Salmonella* colonies were isolated by culture using selective media according to standard protocols outlined by the International Organization for Standardization (ISO 6579:2002). These were further confirmed as *Salmonella* spp. using the matrix-assisted laser desorption-ionization time of flight mass spectrometry (MALDI-TOF MS) direct colony technique.^9^

To summarize the prevalence data of *Salmonella* at each stage, proportions with 95% CIs were computed using R version 4.1.3 software (R Core Team, Vienna, Austria).^10^

## Results and Discussion

Table 1 summarizes the prevalence of *Salmonella* along the value chain nodes.

**Table 1.** *Salmonella* prevalence across the different value chain nodes

Node	Sample	N	Prevalence (95% CI)
**1. Slaughter**	Dressed pig carcasses	144	18.1% (12.3–25.5%)
**2. Carcass transportation**	Meat transportation containers	113	23.9% (16.6–33.0%)
**3. Raw pork retail**	Retail raw pork cuts	247	28.0% (22.6–34.2%)
	Raw pork retail outlets	125	46.4% (37.5–55.5%)
**4. Cooked pork retail**	Cooked pork	104	1.9% (0.3–7.5%)
	Raw vegetable salad	81	8.6% (3.8–17.5%)
	Raw vegetable salad + cooked pork	80	10% (4.7–19.3%)

The prevalence of *Salmonella* on dressed pig carcasses at the slaughterhouses is comparable with that reported by Wilson et al., who examined mesenteric lymph nodes and intestinal contents from slaughter pigs entering this value chain.^3^ This is consistent with studies indicating that infected slaughter pigs raise the likelihood of carcass contamination.^2^ Thus, this emphasizes the need to implement interventions that reduce the entry of infected pigs into the slaughter process, minimize carcass contamination during slaughter and enable decontamination of carcasses before they leave the slaughterhouses.^2^

The contamination of nearly one-quarter of the meat transportation containers before loading freshly slaughtered carcasses underscores the potential risk of cross-contamination of carcasses with *Salmonella* at this particular stage. The highest levels of contamination were observed at the raw pork retail outlets, highlighting this node as a potential source of infection to consumers purchasing pork for household consumption, especially because some pork consumers in this population previously indicated that they frequently eat undercooked pork at home.^11^ The lowest prevalence was observed from cooked pork samples and can be attributed to cooking, which reduces *Salmonella* prevalence via thermal inactivation. The contaminated raw vegetable salads, however, are an illustration of cross-contamination during food preparation, and hygiene messaging could be targeted to cooked pork retailers.

These findings have significant public health implications. In Wilson et al.’s study,^3^ 13 NTS serovars were identified in slaughter pigs within this value chain, including Enteritidis, Hadar, Heidelberg and Uganda, which have been associated with severe gastroenteritis outbreaks worldwide, resulting in illness and even death.^2,12^ The assumption made in this study is that these same serovars are carried along the value chain to the consumer level, thereby making the observed contamination levels a cause for concern.

The increasing demand for pork in this region is outpacing the development of public health infrastructure required to support this growth.^8^ Without adequate mitigation strategies, pork may become an increasingly significant source of NTS infections. Further comprehensive investigations are required to quantify the actual risk posed to pork consumers in this region, assess the contribution of each stage of production to this risk and analyze the behavior of the individuals involved at each stage of the value chain. Such studies are essential for designing targeted interventions aimed at reducing the risk to consumers, particularly considering that this value chain operates in one of Kenya's most densely populated and low-income regions characterized by a high burden of diarrheal diseases and co-endemic pathogens.^13^

### Conclusion

The findings of this study demonstrate the propagation of NTS contamination along the pork value chain in rural western Kenya, thereby highlighting the potential risk to pork consumers and necessitating additional investigations to evaluate this risk and identify influential factors at each stage.

Furthermore, these findings contribute to filling the data gap regarding the incidence of significant foodborne disease hazards in animal source food value chains in developing countries. More of these data are necessary to improve our estimation of the global foodborne disease burden.

## Data Availability

Data are available in an open access format via the open access repository at the University of Liverpool. https://doi.org/10.17638/datacat.liverpool.ac.uk/2401.
